# Training model in abdominal wall endoscopic surgery for ventral hernias. Extended totally extra-peritoneal approach (e-tep)

**DOI:** 10.1590/ACB360808

**Published:** 2021-10-08

**Authors:** Mauricio Andrade Azevedo, Heitor Marcio Gavião Santos, Guilherme Blattner Torres de Oliveira, Murillo de Lima Favaro, Leandro Totti Cavazolla

**Affiliations:** 1 PhD. Department of Gastrosurgery – Universidade Federal de São Paulo (UNIFESP) - Sao Paulo (SP), Brazil; 2 MD. Hernia Center - Hospital São Lucas - Rio de Janeiro (RJ), Brazil.; 3 Msc. Department of Gastrosurgery – Universidade Federal de São Paulo (UNIFESP) - Sao Paulo (SP), Brazil.; 4 PhD. Department of Surgical Technique – Universidade Santo Amaro (UNISA) - Sao Paulo (SP), Brazil.; 5 PhD, Assistant Professor. Department of General Surgery - Universidade Federal do Rio Grande do Sul (UFRS) - Porto Alegre (RS), Brazil.

**Keywords:** Hernia, Ventral, Abdominal Wall, Training Program

## Abstract

**Purpose:**

To develop a reproducible training program model covering the steps of the extended totally extraperitoneal approach (e-tep) technique for correction of ventral or incisional hernia repair.

**Methods:**

Training sessions with surgeons in the laboratory using both porcine specimens and a new ethylene vinyl acetate (EVA) model simulating the operative steps of the e-tep technique. Students were interviewed and asked to answer a questionnaire pre and post the sessions to assess their performance and evaluated the course and model.

**Results:**

A total of 25 trained abdominal wall surgeons was evaluated at the end of the course. It was obtained a 100% satisfaction score of the training, as well as increased confidence levels up to 9 and 10 in all technical aspects of the surgery, having 96% of the surgeons performed a surgery under supervision of the proctors after the course.

**Conclusions:**

This training model is simple, effective, low cost, and replicable in guidance on the beginning of e-tep technique adoption, and performance. As a result, surgeons can get more confident and more able to perform surgeries employing this technique.

## Introduction

It is estimated that in the United States approximately 400,000 incisional hernioplasties are performed each year^1^, with a gradual increase in the number of minimally invasive surgery cases. This type of surgery has been gaining ground, with the advantages of faster recovery and a lower rate of surgical wound infection^2^^-^ 4.

In this context, there is the extended totally extraperitoneal (e-tep) technique, described by Bellyansky *et al.* 5, which consists of an endoscopic repair introducing a mesh in the retromuscular and pre-peritoneal space after closure both of the posterior and anterior aponeuroses managing to bring the rectus abdominis muscles to a more central position and restoring the midline functionality.

Although this is a relatively new technique, it has shown excellent results for ventral hernias, associated or not to the rectus muscle diastasis, and for complex incisional hernias^5^^-^ 7. In the latter, there is the probability for a posterior component separation for the approximation of both posterior and anterior aponeurosis^8^.

The e-tep technique is an advanced repair of the abdominal wall reconstruction, requiring specific training, especially for a better understanding of surgical ergonomics, positioning of the trocars (avoiding injury to the semilunar line and vasculo-nervous bundles), placement of a balloon dissector in the retromuscular space, as well as performing the sutures of the posterior aponeurosis and reconstruction of the midline (anterior aponeurosis), that is done in an inverted suture^5^^-^ 10.

In Brazil, surgery training with human cadaver models is bound by extremely stringent rules^11^. In countries where these rules are less strict, however, training courses are very expensive^12^, and this makes the use of human cadavers impracticable for teaching all the steps of a surgery for more than one student. For these reasons, a training model was proposed in which the essential steps (both theoretical and practical) for a safe performance of the e-tep technique are covered.

## Methods

All procedures and training in this study were in accordance with the ethical standards of the institution research committee and with the 1964 Helsinki Declarations and its latter amendments or comparable ethical standards (CAAE: 38173420.8.0000.5551).

**Figure 1:**
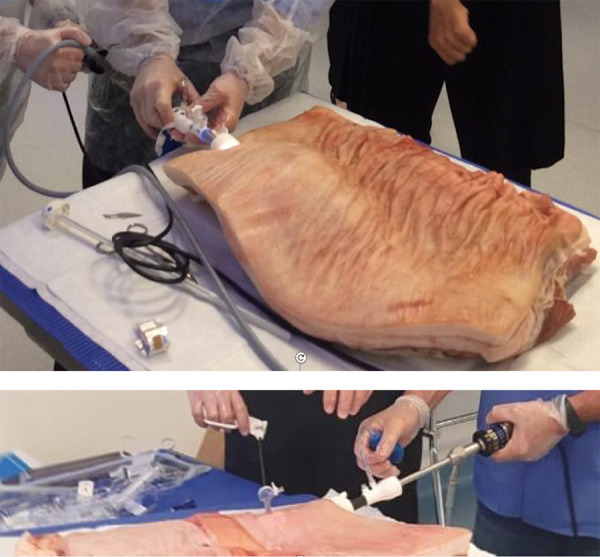
Development of the retromuscular space with a dissection balloon and dissection of both muscular and aponeurotic layers on a porcine belly specimen.

During the period of one year, five training courses containing five surgeons per group were performed. The all 25 surgeons were experts in minimally invasive abdominal wall surgery, but inexperienced in the e-tep technique. This training course was divided in two days, and the first one was divided in four steps. In step 1 a theoretical learning session was held with emphasis on revision of the abdominal wall anatomy focused on the e-tep technique, discussion of cases, radiology images, and the aspects of the step-by-step e-tep technique. In step 2, the students went to a simulation laboratory, where they performed initial operative steps, development of the retromuscular space (with and without a dissection balloon), and dissection of both muscular and aponeurotic layers on a porcine belly specimen (Fig. 1).

**Figure 2:**
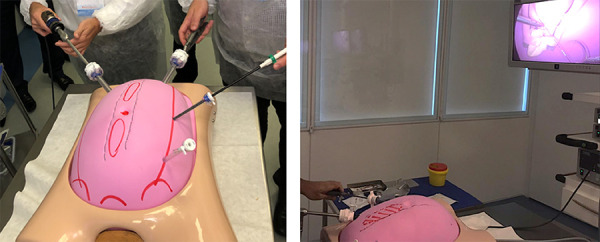
Trocar placement in the ethylene vinyl acetate (EVA) model and simulation of abdominal wall suturing.

**Figure 3:**
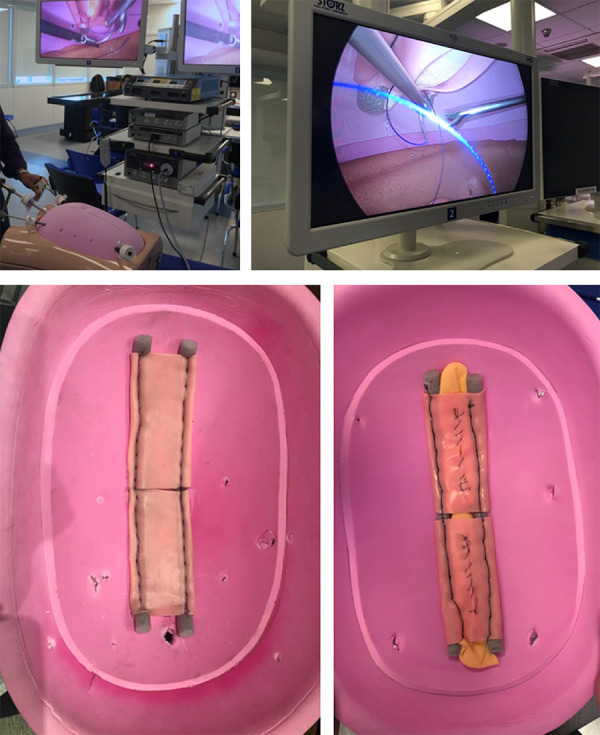
Back-hand suturing simulating the closure of the anterior aponeurosis using a barbed suture in the ethylene vinyl acetate (EVA) model.

In step 3 trocar placement was in a laparoscopic simulator with a semi-rigid ethylene vinyl acetate (EVA) abdominal wall, on which the anatomical landmarks (umbilical region, pubic bone, and costal margins) were previous marked. This enabled forceps triangulation and, consequently, ergonomically better positions during surgery (Fig. 2). In this model two plates, composed of two layers of ethylene vinyl plastic approximately 5-cm wide and 30-cm long, were created and sewn onto the inside of the EVA abdominal wall, supported by two round polyethylene rods with an opening in the medial region running along the length of the wall, thus simulating a hernia defect in the anterior aponeurosis. In this area, which consisted in the step 4 of the training, the students practiced the back-hand suturing, simulating the closure of the anterior aponeurosis using a barbed suture (Fig. 3).

On the second day, the surgeons in training participated as observers in the surgical center of two live surgeries, in which they could interact with the leading surgeons by asking questions and discussing the case.

## Results

A survey was conducted comprising individual interviews and, after training completion, anonymous online questionnaires.

**Figure 4:**
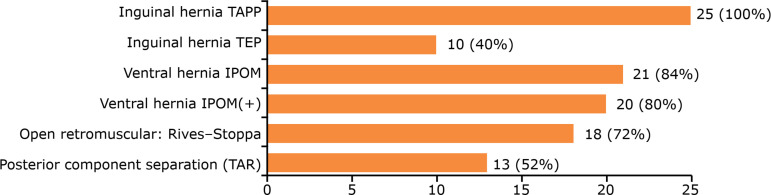
Laparoscopic routine of the surgeons who participated the training.

TAPP: transabdominal preperitoneal; TEP: totally extraperitoneal; IPOM: intraperitoneal onlay mesh with closure of the hernia defect; TAR: transverse abdominal release.

**Figure 5:**
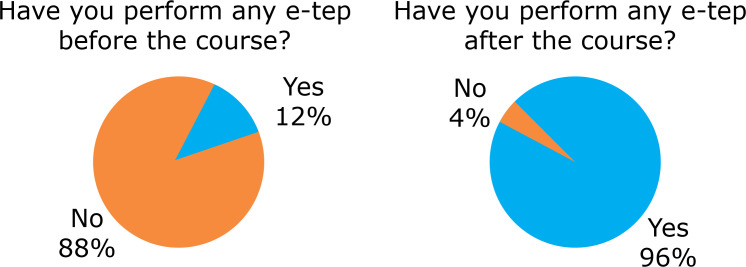
Number of surgeons who had performed e-tep before and after the training course.

All participants had expertise in laparoscopic inguinal hernioplasty, and 80% performed the intraperitoneal onlay mesh with closure of the hernia defect (IPOM) technique for ventral hernia repair (Fig. 4). Among all the participants of the courses, 12% had already tried to perform e-tep technique once (Fig. 5).

e-tep: extended totally extraperitoneal approach.

These surgeons reported being totally satisfied with the program, and that the training model exceeded their expectations, reaching 100%, met all their goals and provided clarification on the e-tep approach (Table 1).

**Table 1:** Feedback at the end of the course.

Theorical learning session	4.4
Laparoscopic simulator with EVA	5
Simulation in laboratory	4.8
Simulation in porcine model	5
Live surgery	5
Discussion of cases and radiology images	4.8
Did the course exceeded the expectation?	Yes 100% No 0%
Would you recommend this course?	Yes 100% No 0%

EVA: ethylene vinyl acetate; 1: poor; 2: not satisfactory; 3: regular; 4: good; 5: excellent.

**Figure 6:**
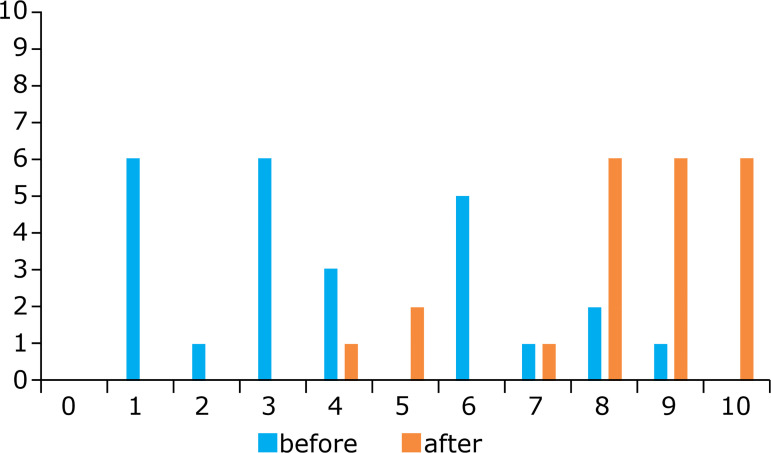
Confidence level before and after the training course for performing e-tep.

Before training, 64% of the students had a confidence level up to grade 4 (0-10 scale) to perform an incisional hernioplasty using e-tep technique, while after the training 64% had a confident level between 9 and 10 (Fig. 6).

e-tep: extended totally extraperitoneal approach.

**Figure 7:**
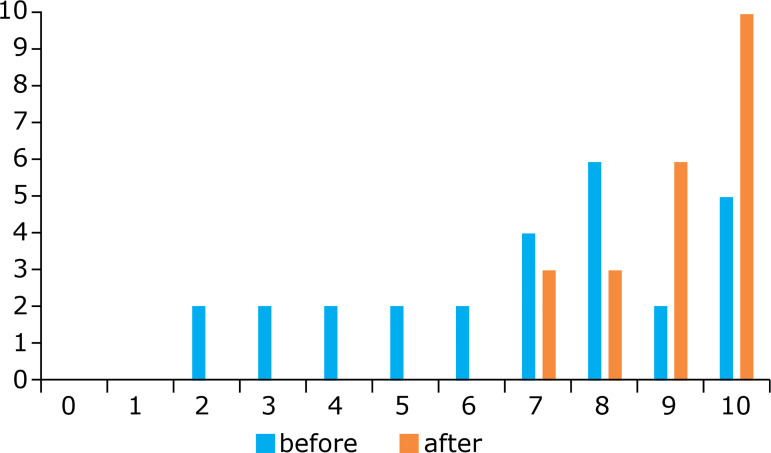
Confidence level before and after the training course for performing back-hand suturing.

When asked about their pre-training confidence level to perform back-hand suturing of the anterior aponeurosis, 48% of surgeons said it was 6 (on the same scale, 1-10). After the training was completed, the lowest level of confidence reported was 7 (13%), and the confidence level of 10 reached 52.2% (Fig. 7).

**Figure 8:**
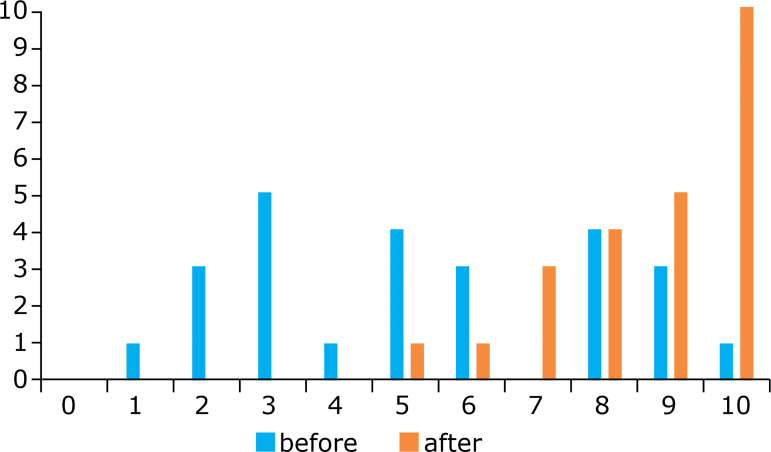
Confidence level before and after the training course to dissect and develop retromuscular and pre-peritoneal space.

Regarding retromuscular and pre-peritoneal space development and the use of a dissection balloon, 52% of the surgeons reported a confidence level that was no higher than 5 before training. Post-training, 17.4% scored it as 8 or 9, and 47.8% rated their confidence level as 10 (Fig. 8).

## Discussion

Ventral herniation, whether in association with rectus muscle diastasis or not, is a frequent condition, and incisional hernioplasty is one of the most common types of surgery performed by general surgeons^3^^,^ 7. Among potential surgery options, retromuscular repair stands out by presenting both lower recurrence and lower surgical wound infection rates^10^. In this context, minimally invasive surgery provides faster recovery, as well as less postoperative pain.

The e-tep technique described by Belyansky^5^ provides safe and lasting repair, and this technique simulates the principles of open surgery, but with lower rates of postoperative pain, infection and recurrence^5^^-^ 7 ^,^ 9.

Specific training in this technique is necessary not only for the surgeons understand the entire anatomy of the abdominal wall, and therefore to be able to navigate between all spaces–the retromuscular (behind the rectus muscle), pre-peritoneal space (subxiphoid and Retzius space area) and the traversalis fascia–, but also to avoid making potentially irreparable damage to neurovascular bundles, which can be a big problem and lead to a failed surgical repair.

One of the most important steps in the laparoscopic e-tep repair is the correct trocar placement, because it provides surgeons with good ergonomic conditions as they work on all abdominal quadrants and also suturing of both the posterior and the anterior wall of the rectus abdominis muscle. So, in this specific training course, it was possible to see the improvement in both basic and advanced suturing skills of the surgeons.

To date, most advanced video-laparoscopy training programs have used in-vivo porcine models or human cadavers^9^^-^ 15, but this makes these courses hard to reproduce, more expensive, and it is necessary a top-quality facility infrastructure, as well a specific logistics^15^^-^ 18. Then, it was described here an educational simulation package containing a surgical training program with porcine specimens and a specific EVA model adding a unique simulation of fundamental e-tep technical skills.

In this training model, the surgeons had the chance to have a realistic experience and significant benefit from a step-by-step approach to the e-tep technique, to practice and simulate retromuscular space development with or without a dissection balloon, with better trocar placement, and to simulate the steps leading to closure of the posterior and anterior aponeuroses of the rectus muscle. As a high level of satisfaction of the trained surgeons was achieved, it is possible to consider that the training had good performance with low cost.

The second day of the course was primordial, because by experiencing live surgeries and being able to interact, asking questions and discussing the cases, the training surgeons had a more realistic view of the surgical steps of the surgery, as well as could witness any pitfalls that can happen during the procedure.

The present study has some limitations, such as a small number of participants, and this can lead to a self-report bias when they evaluated the training program. However, all surgeons answered the questionnaire, and the number of 25 advanced laparoscopic surgeons interested in a specific technique for advanced abdominal wall repair can be a significant number.

## Conclusion

The proposed model training program for teaching minimally invasive hernioplasty with the e-tep technique was found to be efficient and replicable, and therefore it can be widely used as an initial learning tool.
